# Error in Table 3

**DOI:** 10.1001/jamahealthforum.2026.2521

**Published:** 2026-07-10

In the Original Investigation titled “Institutional Special Needs Plans and End-of-Life Outcomes for Nursing Home Residents With Dementia,”^1^ published online on May 31, 2026, there were errors in data in Table 3. For the end-of-life outcomes, mechanical ventilation and enrollment in hospice, the data representing the direct effects of a nursing home’s participation in non-UnitedHealthcare Institutional Special Needs Plans were incorrect. The upper bounds of the 95% CIs were published as negative numbers, but they should have been positive numbers. The negative signs have been deleted from the upper bounds of these 95% CIs. This article has been corrected online.
